# A scoping review of internal hospital crises and disasters in the Netherlands, 2000–2020

**DOI:** 10.1371/journal.pone.0250551

**Published:** 2021-04-26

**Authors:** Vincent W. Klokman, Dennis G. Barten, Nathalie A. L. R. Peters, Marieke G. J. Versteegen, Jaap J. J. Wijnands, Frits H. M. van Osch, Menno I. Gaakeer, Edward C. T. H. Tan, Arjen Boin

**Affiliations:** 1 Department of Emergency Medicine, VieCuri Medical Center, Venlo, The Netherlands; 2 North Limburg Safety Region, Limburg, The Netherlands; 3 Department of Clinical Epidemiology, VieCuri Medical Center, Venlo, The Netherlands; 4 Department of Emergency Medicine, Admiraal de Ruyter Hospital, Goes, The Netherlands; 5 Department of Trauma Surgery and Emergency Medicine, Radboud University Medical Center, Nijmegen, The Netherlands; 6 Department of Political Science, Leiden University, Leiden, The Netherlands; Technion - Israel Institute of Technology, ISRAEL

## Abstract

**Background:**

Internal hospital crises and disasters (IHCDs) are events that disrupt the routine functioning of a hospital while threatening the well-being of patients and staff. IHCDs may cause hospital closure, evacuations of patients and loss of healthcare capacity. The consequences may be ruinous for local communities. Although IHCDs occur with regularity, information on the frequency and types of these events is scarcely published in the medical literature. However, gray literature and popular media reports are widely available. We therefore conducted a scoping review of these literature sources to identify and characterize the IHCDs that occurred in Dutch hospitals from 2000 to 2020. The aim is to develop a systematic understanding of the frequency of the various types of IHCDs occurring in a prosperous nation such as the Netherlands.

**Methods:**

A systematic scoping review of news articles retrieved from the LexisNexis database, Google, Google News, PubMed and EMBASE between 2000 and 2020. All articles mentioning the closure of a hospital department in the Netherlands were analyzed.

**Results:**

A total of 134 IHCDs were identified in a 20-year time period. Of these IHCDs, there were 96 (71.6%) emergency department closures, 76 (56.7%) operation room closures, 56 (41.8%) evacuations, 26 (17.9%) reports of injured persons, and 2 (1.5%) reported casualties. Cascading events of multiple failures transpired in 39 (29.1%) IHCDs. The primary causes of IHCDs (as reported) were information and communication technology (ICT) failures, technical failures, fires, power failures, and hazardous material warnings. An average of 6.7 IHCDs occurred per year. From 2000–2009 there were 32 IHCDs, of which one concerned a primary ICT failure. Of the 102 IHCDs between 2010–2019, 32 were primary ICT failures.

**Conclusions:**

IHCDs occur with some regularity in the Netherlands and have marked effects on hospital critical care departments, particularly emergency departments. Cascading events of multiple failures transpire nearly a third of the time, limiting the ability of a hospital to stave off closure due to failure. Emergency managers should therefore prioritize the risk of ICT failures and cascading events and train hospital staff accordingly.

## Introduction

### Background

Emergency medical services and emergency departments (EDs) within hospitals are community-based resources responsible for the initial medical response to a wide variety of disasters [1]. Hospitals are generally prepared for external events occurring in the community, such as natural disasters and mass casualty incidents [2]. However, hospitals are less prepared for internal events resulting in the (potential) loss of vital medical infrastructural systems [2]. When such an event occurs, we speak of an internal hospital crisis and/or disaster (IHCD), which we define as *a sudden onset event that severely disrupts the everyday*, *routine services of a hospital facility*. In popular parlance, these are also referred to as “major incidents within hospitals”.

The causes of IHCDs can be internal (e.g., information and communication technology (ICT) failure, sabotage, fire), but also external (community-wide events such as natural disasters that affect the services of a hospital) [2]. IHCDs have the potential to threaten the wellbeing of patients and staff (injured persons, casualties) and may cause catastrophic damage and/or total loss of function despite the institution’s full resource mobilization [3–5]. Globally, internal hospital disasters seem to occur with regularity; reported causes include natural disasters [6, 7], toxic substances [8], structural failures [9], fires [10, 11], flooding [12] and terrorist threats [13].

### The Netherlands: Geography and healthcare

The Netherlands is a Western European country with 17.4 million people [14] and has been assessed to be the 16^th^ most exposed country in the world and the most vulnerable country in Europe to natural disasters due to significant below sea-level land area and increasing storm prevalence [15]. However, a sound infrastructure and healthy finances offset these hazards, making the effective rank 65^th^ worldwide [15]. Between 2000 and 2020, the Netherlands suffered seventeen natural hazard-induced disasters and five technologically induced disasters [16]. The most common disasters were storms (10), extreme temperatures (7), fires (2), explosions (1), flooding (1), and railroad accidents (1). These events varied greatly in magnitude but collectively resulted in 2,439 deaths and substantial financial losses [16].

The Netherlands has a modern healthcare system with effective primary care and specialized acute and critical care facilities that are highly ranked and analogous in quality and access to other Western countries [17]. Eighty-three hospitals provide 24-hour emergency care, and 4 additional locations are open during the day and evening hours [18]. The hospital network is subdivided into 8 academic hospitals (tertiary care centers), 26 teaching hospitals (institutions providing specialized care and physician specialty training) [19], and 53 general peripheral hospitals providing general services. Fourteen hospitals serve as level-one trauma centers organized into eleven referral trauma regions [20]. The total number of hospitals has decreased over time due to mergers and centralization of emergency care from 107 acute care hospitals in 2003 to 87 in 2020 [18]. To maintain hospital accreditation, every healthcare institution must maintain a hospital disaster plan, and since 2005, yearly training has been required in accordance with this plan [21].

### Scope and objectives

Little is known about the frequency and type of IHCDs in the Netherlands. Furthermore, IHCDs are scarcely reported in the medical literature, as hospitals rarely report on single events. In contrast, newspapers, news press releases and other gray literature often publish valuable information on these incidents. A scoping review strategy was chosen to acquire all available reports of IHCDs in the medical literature, gray literature and news media in Dutch hospitals from 2000 until 2020. Cataloging and analyzing these results would allow healthcare institutions to increase hospital disaster preparedness and improve business continuity in times of crisis. The objective of this study was to map the evidence of IHCD occurrence, identify key characteristics and factors related to IHCDs, and assess future risk and temporal trends in IHCD occurrence.

## Materials and methods

A scoping review was conducted following the framework described by Arksey & O’Malley [22] and reported per PRISMA-ScR guidelines [23]. The scoping review protocol was developed *a priori* to guarantee reproducibility and transparency. The protocol, incident definitions, data characterization form, repository of all relevant articles and dataset are available upon request. The incident review team consisted of individuals with multidisciplinary expertise in emergency medicine, public health, epidemiology, crisis analysis and crisis management. The institutional review board of VieCuri Medical Center approved this research project (#439).

### Search

Public search engines (PubMed, Medline, Google, Google News) and the LexisNexis newspaper article database were used to search for articles and press releases in the Netherlands between January 1, 2000, and December 31, 2019. The search was conducted in January of 2020. The search terms “hospital,” “closed,” “ICU,” “ED,” “department,” “failure,” “fire,” and “evacuation” and their synonyms were combined with Boolean operators (S1 Appendix).

### Article selection

Articles were only included in this study if one or more departments or intervention units (operating rooms (ORs), diagnostic rooms/equipment) of Dutch hospitals suddenly and unexpectedly had to be closed, were evacuated and/or if injured persons needed treatment. Events were excluded if inpatient or critical care departments were unaffected or if the event did not take place at a full-fledged hospital (i.e., hospitals containing an ED and ICU). Partial closures and partial evacuations were excluded. Hospital or ED closures due to overcrowding, staffing shortages, staff strikes, nationwide epidemics, medical malpractice, and insect manifestations were also excluded. The search was conducted by two of the authors (V.W.K. and D.G.B.). Duplicates were manually removed. When discrepancies could not be resolved regarding inclusion and characterization, three other reviewers made the final judgement (M.G.J.V., N.A.L.R.P., M.I.G.).

### Event classifications

Each event was categorized based on the cause of hospital department closure. A cascade of failures (or concurrent malfunctions) are single failures occurring in a complex and interconnected network, which can lead to the complete fragmentation of a system [24]. In cascading events, the events were sequentially noted, with the initial instigating event considered the primary cause utilized in this analysis. Each of the events was classified into technical failures, ICT failures, fires (internal and external), power failures (internal and external), hazardous materials, structural failures, utility failures, loss of medical gases, hydrometeorological, security and violence. Fire and power failures were further categorized based on location as they can either arise from within the hospital terrain or externally from an outside source or provider. S2 Appendix details the exact classification definitions.

### Analysis and data charting

All articles mentioning the closure of a hospital were downloaded and data were extracted and independently characterized. Collected data included: location, temporal aspects, failure types, casualties, departments involved, evacuation, patient presentation stops, injuries and casualties. All collected data were exported into Excel spreadsheets (Microsoft Corporation, Redmond, WA) and analyzed descriptively. Hospital demographics regarding bed size and trauma level were retrospectively ascertained from the Dutch Public Healthcare Database [18] to provide perspective on the impact of each IHCD and its respective affected hospital. Hospital type and trauma levels in the Dutch context are explained in S2 Appendix. IHCD risk per institution was assessed by dividing IHCD incidence per year by the number of hospitals.

## Results

### Selection of sources of evidence

**Fig 1:**
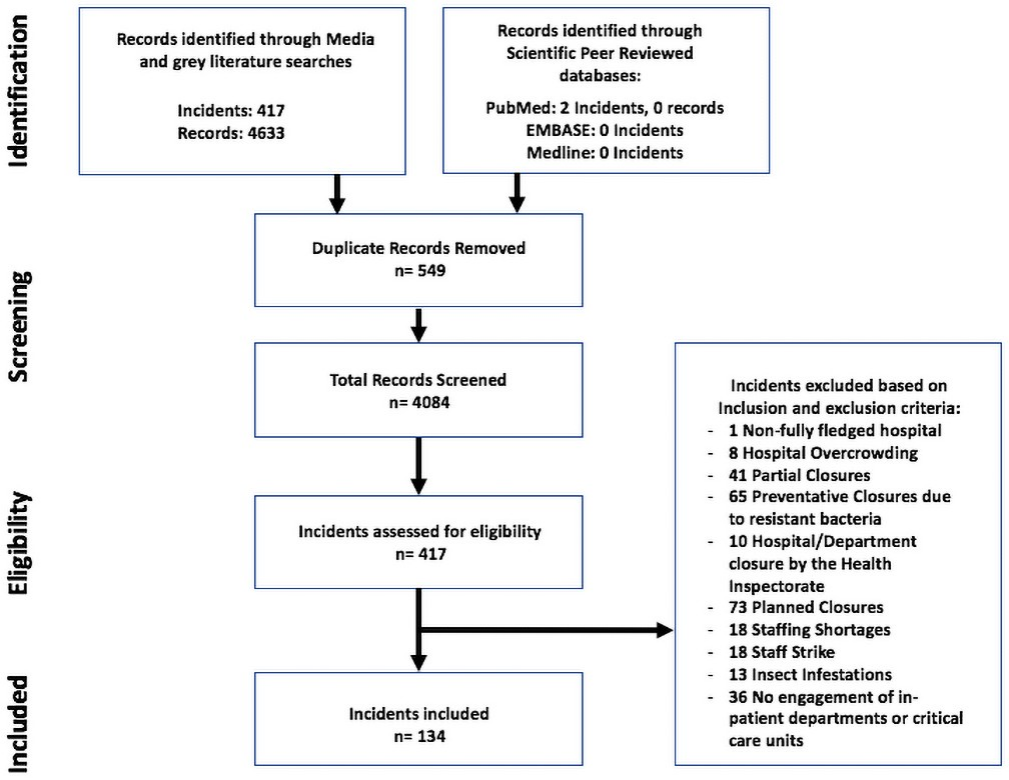
Flow diagram of the scoping review process. Process during the screening and identification of internal hospital crises and disasters in the Netherlands between 2000 and 2020.

A total of 4,633 records mentioned the closure of a hospital or hospital department. After screening and application of eligibility criteria, a total of 4,084 records identified 134 IHCDs (Fig 1).

### Scoping review descriptive statistics

**Fig 2:**
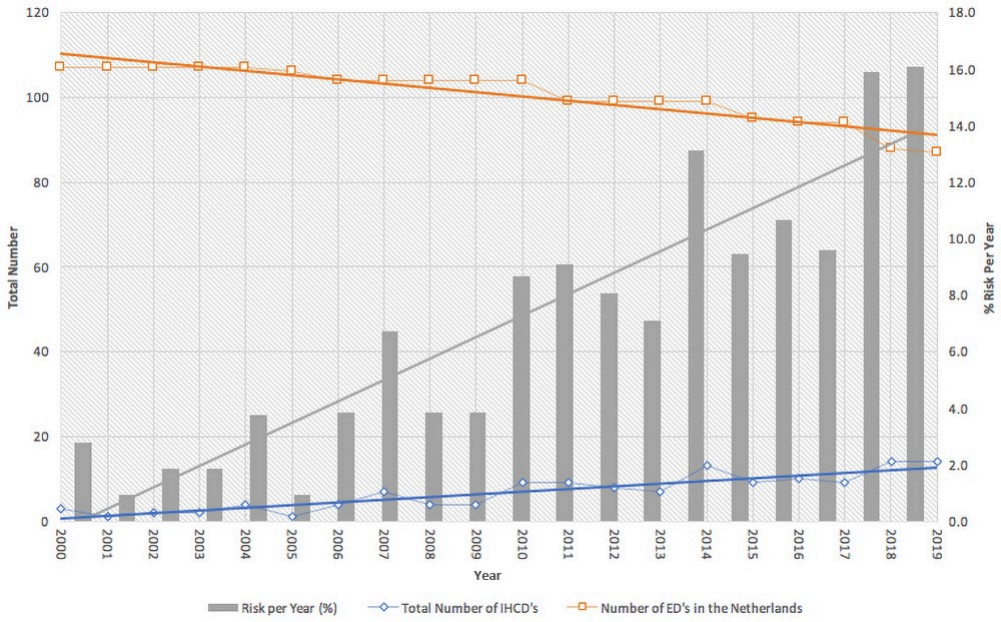
Number of emergency departments plotted against the total number of internal hospital crisis and disasters. The calculated risk (in %) is depicted per year, showing an increasing trend.

**Fig 3:**
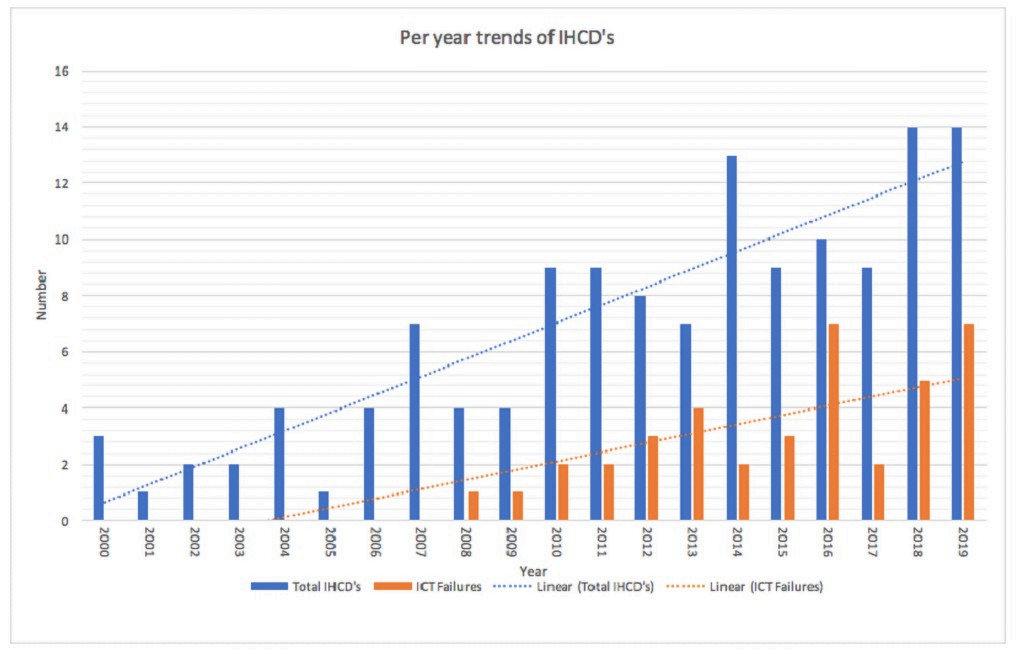
Distribution of total internal hospital crises and disasters and primary ICT failures. Occurrence in the Netherlands by year: 2000–2019 with a linear trend over time.

IHCDs occurred at a mean of 6.7/year in 61 different hospitals. Fig 2 depicts the number of incidents per year and the risk of IHCD occurrence per hospital and shows an increasing trend. This resulted in an average risk of 6.9%, which ranged from 2.8% in 2000 to 16.1% in 2019. A linearly increasing trend in IHCDs was observed (Fig 2), with a mean rate of 10.2 IHCDs per year in the latter 10 years (2010–2019) compared to 3.2 IHCDs per year in the initial 10 years (2000–2009). In the initial decade there was one primary ICT failure, which compared to 32 primary ICT failures in the latter decade. Fig 3 shows the absolute number of IHCDs and the total number of ICT failures with linear regression.

Every Dutch province experienced an IHCD with the majority of IHCDs occurring in the more populous regions of North Holland 33 (24.6%) and South Holland 32 (23.9%), where there is an average of >10 hospitals within a 20-km distance [25]. All types of hospitals were affected by IHCDs, including academic hospitals and level one trauma centers. The general hospital characteristics are depicted in Table 1.

**Table 1:** Hospital characteristics of internal hospital crises and disasters (IHCDs) occurring in Dutch hospitals from 2000 to 2019.

Characteristics and Categories	Total number of Incidents, %, N = 134
Type of Hospital*	
Academic	20 (14.9)
Teaching	68 (50.7)
Peripheral	46 (34.3)
Trauma Level*	
1	31 (27.4)
2	74 (55.2)
3	29 (21.6)
**Hospital Bed Size (No. of Beds)**	
<400	34 (27.4)
400–600	39 (29.1)
>600	61 (45.5)

* Hospital classification and trauma level designations are explained in S2 Appendix.

Of the 134 IHCDs, 108 (80.6%) were isolated internal IHCDs, while 26 (19.4%) were combined internal and external events that eventually affected the internal functioning of the medical facility. The primary causes of IHCDs were ICT failures (n = 33; 24.6%), technical failures (n = 29; 21.6%), fires (n = 24; 17.9%), power failures (n = 21; 15.7%), hazardous materials (n = 15; 11.2%), structural failures (n = 5; 3.7%), meteorological (n = 4; 3.0%), and security and violence (n = 3; 2.2%).

Event cascades were prevalent in 39 (29.1%) IHCDs, resulting in multiple failure type classifications being applied to a single IHCD (Table 2). Cascading events resulted in a total of 193 failures (Table 2), especially evident in internal power failures, as they accounted for 3.0% of primary failures, compared to 11.9% of total failures.

**Table 2:** Internal hospital crises and disasters (IHCDs) characteristics in failure type, localization and cascading events of IHCDs occurring in Dutch hospitals from 2000 to 2019.

**Characteristics and Categories**	**Primary Failures, %, N = 134**	**Total Failures, %, N = 193**
Technological Failure	29 (21.6)	45 (23.3)
ICT Failure	33 (24.6)	39 (20.2)
Internal Fire	22 (16.4)	27 (14.0)
External Fire	2 (1.5)	2 (1.0)
Internal Power Failure	4 (3.0)	23 (11.9)
External Power Failure	17 (12.7)	18 (9.3)
Hazardous Materials	15 (11.2)	19 (9.8)
Structural Failure	5 (3.7)	7 (3.6)
Utilities Failure	0 (0.0)	4 (2.1)
Loss of Medical Gasses	0 (0.0)	2 (1.0)
Meteorological	4 (3.0)	4 (2.1)
Security & Violence	3 (2.2)	3 (1.6)
**Type of IHCD**	**Count, %, N = 134**	**Count, %, N = 39**
Internal	108 (80.6)	
Combined	26 (19.4%)	
**Cascading Events**	39 (29.1)	
External power failure: technical failure: internal power failure		9 (23.1)
ICT failure after power failure		6 (15.4)
Technical failure leading to fire		4 (10.3)
Technical failure leading to internal power failure		4 (10.3)
Technical failure leading to hazardous material incident		3 (9.0)

Out of 134 IHCDs, a total of 96 (71.6%) ED closures, 76 (56.5%) operation room (OR) closures, and 56 (41.8%) evacuations were identified (Table 3). Of the 56 total evacuations, 37 (66.1%) patients/staff/visitors were displaced internally, 12 (21.4%) IHCDs caused the evacuation of patients externally, and 7 (12.5%) events patients were evacuated both internally and externally. These IHCDs caused major disruptions in the hospital critical care departments, with 110 events (82.1%) having ED involvement. Of these cases, the ED was directly affected by the IHCD in 96 IHCDs (87.2%) and activated for the care of injured patients in 26 IHCDs (23.6%). In 26 (17.9%) of the incidents, there were injured people; in 2 (1.5%) incidents, there were casualties (2 total casualties).

**Table 3:** Departmental effects, evacuation locations and injuries of IHCDs occurring in Dutch hospitals, 2000 to 2020.

Characteristics and Categories	Total, %, N = 134	Total, %, N = 56
ED Involvement^a^	110 (82.1)	
ED Presentation Stop	96 (71.6)	
Operating Room Stop	76 (56.7)	
Evacuations	56 (41.8)	
• Internally		37 (66.1)
• Externally		12 (21.4)
• Combined		7 (12.5)
Injuries	24 (17.9)	
Casualties	2 (1.5)	

^a^Emergency department (ED) involvement includes as incidents directly involving the ED, incidents affecting surge capacity (i.e., injuries) and causing patient presentation to stop at an ED.

## Discussion

The study showed that hospital and departmental closures occurred 134 times in the Netherlands, with an increasing trend across a 20-year study period. This increase is associated with a higher number of ICT failures. This is a notable trend, as hospitals, and their EDs in particular, serve an important function in the community as wardens of public health in times of crisis and disaster [26]. IHCDs can directly cause physical harm, and the sudden closure of a hospital or ED may force the diversion of patients to distant hospitals where the patients’ records are lacking [27]. Natural disasters rarely occur in the Netherlands, and only four incidents were caused by inclement weather. Multiple sequential failures (cascading events) occur 29.1% of the time when ultimately a hospital or department becomes disabled. EDs are often involved both directly and indirectly, with 71% of IHCDs incurring ED closure.

### ICT failures are an increasing trend

The 20-year-period studied showed an average of 6.7 IHCDs per year. In particular, this study has shown that the incidence is increasing and is in large part explained by the increase in ICT failures. This should not come as a surprise considering that the healthcare sector has seen increases in the use of digital technologies in communication, diagnostics, and treatment applications [28]. The frequent occurrence exposes a potential weakness in hospital preparedness and business continuity that must be addressed. While the search did not uncover any reports of cyberattacks on hospital networks, there is previous experience with ransomware affecting hospital networks in the United Kingdom resulting in multiple, prolonged ED closures [29]. The increasing trend in ICT disturbances makes cybersecurity and digital continuity issues that should be prioritized in hospital disaster preparedness [30]. In the wake of the 2019 Novel Coronavirus (COVID-19) pandemic, E-health and telemedicine have become especially important for doctors to continue delivering care to patients [31], making this susceptible to failure. Furthermore, our data corroborate a recent Dutch governmental report detailing an increasing trend of hospital ICT failures and their potential impacts on patient safety [32].

### Cascading events

Interdependence within complex networks, especially in critical infrastructure networks, increases cascading failure risk and has important implications for infrastructure reliability and security [33]. Hospitals are reliant on external networks while being complex organizations and structures with convoluted interrelated systems themselves [34]. Our data show that hospital resilience in mitigating internal crises appears to be habitually challenged by multiple consecutive failures in the form of cascading events. Nearly one-third of IHCDs express this trend (Table 2). Hospitals customarily seem to be able to absorb one “hit” to their internal infrastructure, such as a simple external power failure where the auxiliary power seamlessly starts and the hospital continues to function. Conversely, if somewhere in that succession of events an additional failure, such as a technological failure (i.e., short circuit) hinders emergency power sources, a hospital fails to be able to provide lifesaving services. Power outages often lead to failures involving computer networks, and technological failures also tend to lead to equipment malfunctions that in turn lead to fires and hazardous material releases. Knowledge of these predominantly occurring sequential events is beneficial for attenuating of safety checks in disaster preparation plans. Examples would include frequent auxiliary power tests, training of personnel to bypass problems with power and the creation of emergency procedure guides in the face of ICT and other essential utilities (e.g., medical gases, water) failure. Furthermore, considering the current high dependence on ICT systems for patient care, the incorporation of double or even triple modular redundancy could prevent failure altogether.

### Hazardous materials

Hospitals are fertile grounds for disaster, as they are packed with flammable gases, toxic substances, biological sources and radioactive materials. Patients injured by these same types of substances ideally present to the ED in a decontaminated state. However, this is not always the case, endangering hospital employees or prompting evacuation. Hospital evacuations occurring from both threatened and actual hazardous material exposure or release typically come with little warning or time to prepare, often occurring in EDs [35]. Between 1971 and 1999, 18% of U.S. hospital evacuations were due to hazardous materials [36]. Comparatively, 19 incidents were found to have occurred in the Netherlands in the past 20 years, each necessitating evacuation (33.9% of total evacuations). High-consequence infectious diseases and emerging infectious diseases also pose significant risks to hospitals and EDs, where hospitals need to be able to safely treat infected individuals while maintaining surge capacity [37, 38]. Unprepared hospitals receiving these types of patients lead to dangerous situations, panic and eventual evacuations. The dataset entails two ED closures lasting several hours due to possible Ebola viral disease infection. Unpreparedness and no prior notice of patient arrival caused considerable delay, while the emergency services scrambled to collect appropriate protective equipment and protocols [39, 40].

### Advice to ED and hospital

Hospitals shelter a high number of vulnerable and dependent individuals, sustained by high densities of medical and support staff. Risks and hazards to potential disasters must be made apparent and planned for. As part of a hospital’s accreditation in the Netherlands, a hospital disaster plan must be available, and the staff must be trained to act in extenuating circumstances to both external and internal sources of disruption [41, 42]. The most common types of internal disaster plans only encompass computer system failures, power failures, and fires [43]. The possible types and trend of IHCDs having transpired in the Netherlands have been enumerated, which can be extrapolated and applied as potential hazards to the majority of modern healthcare facilities in developed countries. If these internal disaster plans are revised in line with these findings and with multidisciplinary input, this will have the potential to improve hospital recovery times and possibly prevent unnecessary closure. Moreover, it is advised to create generic crisis response templates in the form of action cards based on the most common disasters and cascading events. These should incorporate the following elements: establishing the scope of the problem, coping with local consequences, protection against collateral damage, addressing administrative issues, and preparing for problem solution or prolonged failure [34]. This will allow hospital coordinators to create exercises to show employees how the various hospital systems are organized and how to respond to specific failures [34]. These action cards should also be made to address isolated internal and combined external and internal scenarios. Acute and critical care services are often disrupted by IHCDs. These services are the result of a complex, logistical chain within hospitals, where failure may be determined by the weakest link. For example, when an OR and/or ICU are no longer functional, it may be decided to close the ED as a precaution. Therefore, it may be valuable to develop regional agreements between institutions in case of loss of acute care services, especially when greater distances between critical access hospitals are present. Hospital staff should be frequently trained for the occurrence of cascading events (sequential loss of essential services) and scenarios where patient data suddenly become unavailable as a result of ICT failure. Furthermore, cybersecurity awareness should receive the highest priority. The security technique emphasized most often in the literature is proper employee training, because most security breaches are caused by employees accessing malicious files. These breaches are generally not stopped by ICT security systems [21]. Finally, a unified body that registers IHCDs (inter)nationally and that acts as a hospital disaster preparedness expertise center would be a valuable medium where learned lessons can be shared with other medical centers.

### Limitations

There are several limitations in this scoping review. Under ascertainment may have occurred due to the subjective nature of press and news releases; bias towards sensationalistic and newsworthy events may have arisen, leaving out smaller and less impactful incidents. However, it is likely that events in which a critical care or inpatient department had to be closed urgently would have made the news. Underreporting of incidents may have occurred in earlier years due to inaccessibility of articles and decreased number of reporting news outlets (i.e., now more online news articles than print versions). Furthermore, only acute care hospitals were included, and the results cannot be applied to other types of health care facilities. Potentially valuable information on the exact duration of department closure was not available in all incidents. Nonetheless, the sources and databases used are the best available. This study was geographically limited to the Netherlands; however, the incidence and failure types are likely to be applicable to all modern hospitals, with the exception of natural disasters, which are more common in other regions of the world. Additionally, statistical analyses that were performed were univariate, so confounding by other factors cannot be ruled out when interpreting the presented results.

## Conclusion

Healthcare facilities are vulnerable to IHCDs regularly occurring in the Netherlands and have marked effects on hospital critical care departments, EDs in particular. An increasing trend in incidence is observed, which is associated with an increase in ICT failures. Cascading events of multiple failures transpire nearly a third of the time, thus limiting the ability of a hospital to stave off closure due to failure. In order to prevent unnecessary hospital closure, emergency managers should adapt their current hospital disaster plans to include the incidents enumerated in this study, with special attention towards cascading events and ICT failures. Hospital staff should receive regular training with crisis response templates in the form of action cards to respond to both IHCDs and external scenarios.

## Supporting information

S1 Dataset Dataset describing 134 IHCDs from 2000–2020. (XLSX)

S1 Appendix Search strategy implanted to identify IHCDs. (DOCX)

S2 Appendix Definitions and classifications of IHCDs, events and hospital designations. (DOCX)

S1 Checklist Preferred Reporting Items for Systematic reviews and Meta-Analyses extension for Scoping Reviews (PRISMA-ScR) checklist. (DOCX)
