# Supplementary material for: A scoping review of internal hospital crises and disasters in the Netherlands, 2000–2020
Source: PLoS One. 2021 Apr 26;16(4):e0250551. doi: 10.1371/journal.pone.0250551 (PMC8075216; doi:10.1371/journal.pone.0250551)
Supplement: S1 Appendix — (DOCX) [file pone.0250551.s002.docx]

**Appendix 1. Search strategy.**

**Search Strategy:**

1. Medical Literature Search: Pubmed
2. Medical Literature Search: EMBASE
3. Medical Literature Search MEDline
4. Google Scholar
5. Google News
6. General Google Search
7. Lexis Nexis Search

**1. Pubmed:**

Search:

('internal hospital disaster' OR (internal AND (hospital) AND (disaster))) AND (The Netherlands OR Netherlands OR Holland)

Filter since 2000

Hits 197

Relevant: 2

- Barten, D. G., Veltmeijer, M. T., & Peters, N. A. (2019). Emergency Department Ceiling Collapse: Response to an Internal Emergency. Disaster medicine and public health preparedness, 1-2.
- Biesheuvel, T. H., Brouwers, C., & Bloemers, F. W. (2017). Lessons learned from the evacuation of the VU University Medical Centre after flooding. Nederlands tijdschrift voor geneeskunde, 161, D861-D861.

**2. EMBASE**

Search: ('internal hospital disaster' OR (internal AND ('hospital'/exp OR hospital) AND ('disaster'/exp OR disaster))) AND ('netherlands'/exp OR netherlands)

Hits: 56

Relevant: 0

Search: ('hospital disaster' OR (('hospital'/exp OR hospital) AND ('disaster'/exp OR disaster))) AND ('netherlands'/exp OR netherlands)

Hits: 791

Relevant: 0

**3. MEDline**

Internal Hospital Disaster AND Netherlands

Hits: 0

Hospital Disaster AND Netherlands

Hits: 0

**5. Google News & General Google Search & Lexis Nexis**

Filter: Netherlands

Filter: 01-01-2000 until 31-12-2019

**Search terms (Dutch)**

• "Ziekenhuis" en "gesloten" en "storing”

• “Ziekenhuis” en “dicht”

• “Ziekenhuis” en “gesloten”

• “SEH" en “gesloten”

• “SEH” en “dicht”

• “Spoedeisende hulp” en “gesloten”

• “Spoedeisende hulp” en “dicht”

• "Eerste hulp" en “gesloten”

• "Eerste hulp" en “dicht”

• "EHBO" en “gesloten”

• "EHBO" en “dicht”

• “EHH” en “gesloten”

• “EHH” en “dicht”

• “Eerste hart hulp” en “gesloten”

• “Eerste hart hulp” en “dicht”

• “SEH” en “storing”

• “Spoedeisende hulp” en “storing”

• “Ziekenhuis” en “storing”

• “Intensive Care” en “gesloten”

• “Intensive Care” en “dicht”

• “IC” en “Gesloten”

• “IC” en “dicht”

• “Ziekenhuis afdeling” en “gesloten”

• “Ziekenhuis afdeling” en “dicht”

• “Ziekenhuis” en “gesloten” en “brand”

• “Spoedeisende hulp” en “gesloten of dicht” en “brand”

• “SEH” en “dicht” en “ziekenhuis” dicht

• "Ziekenhuis" en "gesloten" en "wateroverlast"

• "Ziekenhuis" en "dicht" en "wateroverlast"

• “Ziekenhuis” en “Ontruimd”

• “Ziekenhuis” en “evacuatie”

• “Ziekenhuis” en “geëvacueerd”

• “SEH" en “ontruimd”

• “SEH" en “evacuatie”

• “SEH" en “Geëvacueerd”

• “Spoedeisende hulp” en “ontruimd”

• “Spoedeisende hulp” en “evacuatie”

• “Spoedeisende hulp” en “Geëvacueerd”

• “Eerste hulp” en “Ontruimd”

• “Eerste hulp” en “evacuatie”

• “Eerste hulp” en “Geëvacueerd”

• “Intensive Care” en “Ontruimd”

• “Intensive Care” en “evacuatie”

• “Intensive Care” en “Geëvacueerd”

• “IC” en “ontruimd”

• “IC” en “evacuatie”

• “IC” en “Geëvacueerd
