# Supplementary material for: A scoping review of internal hospital crises and disasters in the Netherlands, 2000–2020
Source: PLoS One. 2021 Apr 26;16(4):e0250551. doi: 10.1371/journal.pone.0250551 (PMC8075216; doi:10.1371/journal.pone.0250551)
Supplement: S2 Appendix — (DOCX) [file pone.0250551.s003.docx]

**Appendix 2: Definitions and Classifications**

**Table 1. Classification designations of events**

| **Technical failures** | Errors of technology based systems |
| --- | --- |
| **ICT failures** | Any error in telephone, internet, computer based systems |
| **Hazardous materials** | Any item or agent (biological, chemical, radiological, and/or physical), which has the potential to cause harm to humans. High consequence infectious diseases are included in this designation. |
| **Structural failure** | Loss of the load-carrying capacity of a component or member within a structure |
| **Fire** | Combustion of materials causing smoke and heat |
| **Utilities failure** | Disruption of the gas, and/or water supply |
| **Power failure** | Disruption of electricity supply |
| **Loss of medical gasses** | Disruption of supply of essential medical gasses |
| **Hydrometerological** | Transfer of water and energy between the land surface and the lower atmosphere |
| **Security & Violence** | The use of physical force so as to injure, abuse, damage, or destroy property or person. Terroristic events are included in this designation. |

**Table 2. Internal Hospital Disaster Classification**

| **Disaster Main-type** | **Disaster Sub-type** | **Disaster Sub-sub Type** |
| --- | --- | --- |
| Technical Failure | Medical Equipment failure | - Electronic Equipment  - Diagnostic Equipment  - Surgical Equipment  - Storage & Transport Equipment |
|  | Hospital Mobility Failures | - Elevators  - Pneumatic transport system |
| Utility Failure | Power Outage | - Power Failure  - Back-up power supply failure |
|  | Water Failure |  |
|  | Sewer Failure |  |
|  | Loss of Medical Gases |  |
|  | Loss of Heating |  |
|  | Loss of Air Conditioning |  |
| Structural Failure | Loss of structure strength |  |
|  | Loss of Integrity | - Structural fatigue  - Corrosion |
|  | Manufacturing errors | - Improper selection materials  - Incorrect sizing  - Improper heat treating  - Poor workmanship  - Failure of adherence to building design  - Vandalism, sabotage |
| Fire* | Combustible Materials |  |
|  | Flammable liquids |  |
|  | Flammable gasses | - Medical gasses  - Heating gasses  - Cooking gasses |
|  | Flammable metals | Alkali metals (Batteries) |
|  | Cooking oils and fats | Kitchen Fires |
|  | Electrical fire |  |
| Hazardous Materials | Contaminated Person | - Gas forming substances  - High Isolation requiring disease (ebola, smallpox) |
|  | Contaminated Materials | - Mail/packaging  - Building materials  - Pharmaceutical agents  - Laboratory reagents |
|  | Chemical Spill | - Medical Gas Leakage  - Laboratory/Pathology reagents  - Cleaning agents |
|  | Radiation Leakage | - Nuclear Medicine Storage |
| Information and Communication Technology Failure | Computer Failure | - Software Failure  - Hardware Failure  - Patient information system failure  - Cyberattack |
|  | Internet Failure |  |
|  | Failure in Phone services |  |
| Violence | Mass (Terrorist) Attack | - Bomb  - False Alarm (bomb, fire)  - Ransomware |
|  | Interpersonal Violence | - Physical Violence  - Hazardous Materials |
|  | Self-directed Violence | - Physical Violence  - Shooting  - Stabbing  - Hazardous Materials |

*European standards

**Table 3. External Hospital Disaster Classification***

| **Disaster Generic Group** | **Disaster Group** | **Disaster Main-Type** | **Disaster Sub-type** | **Disaster Sub-sub Type** |
| --- | --- | --- | --- | --- |
| Natural Disaster | Geophysical | Earthquake | Ground Shaking |  |
|  |  |  | Tsunami |  |
|  |  | Volcano | Volcanic Eruption |  |
|  |  | Mass movement (dry) | Rock fall |  |
|  |  |  | Avalanche | - Snow Avalanche  - Debris Avalanche |
|  |  |  | Landslide | - Mudslide  - Lahar  - Debris Flow |
|  |  |  | Subsidence | - Sudden Subsidence  - Long-lasting subsidence |
|  | Meteorological | Storm | Tropical Storm |  |
|  |  |  | Extra-tropical Cyclone (winter storm) |  |
|  |  |  | Local/Convective Storm | - Thunderstorm/Lightning  - Snowstorm/blizzard  - Sandstorm/dust storm  - Generic (severe) storm  - Tornado  - Orographic storm (strong winds) |
|  | Hydrological | Flood | General (river) flood |  |
|  |  |  | Flash flood |  |
|  |  |  | Storm surge/coastal flood |  |
|  |  | Mass movement (wet) | Rock fall |  |
|  |  |  | Landslide | Debris flow |
|  |  |  | Avalanche | - Snow Avalanche  - Debris Avalanche |
|  |  |  | Subsidence | - Sudden Subsidence  - Long-lasting subsidence |
|  | Climatological | Extreme temperature | Heatwave |  |
|  |  |  | Coldwave | Frost |
|  |  |  | Extreme winter conditions | - Snow pressure  - Icing  - Freezing rain |
|  |  | Drought | Drought |  |
|  |  | Wildfire | - Forest fire  - Land fire (grass, scrub, bush, etc.) |  |
|  | Biological | Epidemic | - Viral Infectious Diseases  - Bacterial Infectious Diseases  - Parasitic Infectious Diseases  - Fungal Infectious Diseases  - Prion Infectious Diseases |  |
|  |  | Insect Infestation | -Grasshopper/ locust/ worms  - Animal Stampede |  |
| Manmade | Nuclear and Radiation | Lethal effects to individual |  |  |
|  |  | Radioactive isotope to environment |  |  |
|  |  | Reactor core melt |  |  |
|  |  | Radiotherapy accidents |  |  |
|  | Chemical Spill | Simple spill |  |  |
|  |  | Complicated spill |  |  |
|  | Violence | Collective violence | Warfare |  |
|  |  |  | Terroristic Attacks |  |
|  |  |  |  |  |
|  | Infrastructural disaster | Loss of medical supplies |  |  |
|  |  | Loss of financial infrastructure |  |  |

* Natural disasters adapted from: Below, R., Wirtz, A., & Guha-Sapir, D. (2009). *Disaster category classification and peril terminology for operational purposes* (No. UCL-Université Catholique de Louvain).

**Table 4. Dutch hospital classification:**

| Academic Hospital: | Dutch academic hospitals are hospitals affiliated with universities. They are typically larger in size and have multiple tertiary hospital functions. |
| --- | --- |
| Teaching Hospital: | While teaching hospitals may also have tertiary hospital functions, they provide intermediate complex hospital care while also training healthcare professionals. |
| General Peripheral hospitals: | Deliver basic hospital care but generally do not maintain tertiary hospital functions |

**Table 5. Trauma level classifications*:**

| Level 1: | Comprehensive regional tertiary care facility capable of providing total care for every aspect of injury; 24 hours a day. First choice of prehospital providers to refer multiple injured persons; referral hospital for Level 2 and 3 trauma centers.; |
| --- | --- |
| Level 2: | Able to initiate definitive care for all injured patients, though lacking tertiary care capabilities; |
| Level 3: | Ability to provide basic assessment, resuscitation, surgery, intensive care and stabilization of injured patients and emergency operations. Complex cases are usually referred to Level 1 and 2 centers. |

* Adapted from: American Trauma Society [Internet]. (2014). Trauma center levels explained. Available from: [https://www.amtrauma.org/page/traumalevels#:~:text=The%20different%20levels%20(ie.,both%20Adult%20and%20Pediatric%20facilities](http://?#:~:text=The%20different%20levels%20(ie.,both%20Adult%20and%20Pediatric%20facilities).
